# Complicated Amyand’s Hernia in a Neonate

**Published:** 2014-07-10

**Authors:** Parkash Mandhan, Talal Al Rayes, Mansour J Ali, Mahmoud Aldhaheri

**Affiliations:** Department of Pediatric Surgery, Hamad Medical Corporation Doha, Qatar; 1 Department of Surgery, Hamad Medical Corporation Doha, Qatar

**Keywords:** Amyand’s Hernia, Acute Appendicitis, Inguino-scrotal swelling, Neonate

## Abstract

Amyand’s hernia is a rare clinical entity in which the vermiform appendix is present within the inguinal hernia sac. Here, we report a 5-day-old neonate with dysmorphic features referred to us with a tender irreducible right inguino-scrotal swelling. Surgical exploration showed gangrenous appendix with a peri-appendicular abscess in the inguinal hernia sac. Appendectomy and right herniotomy was performed.

## INTRODUCTION

Amyand’s hernia (AH) is rare with a reported incidence of 0.07-0.28% of all cases of inguinal hernia [1]. The presentation of AH is similar to that of any inguinal hernia, with erythema, tenderness and inability to reduce the contents, if hernia is incarcerated. When contained appendix is inflamed, it can too mimic testicular inflammation or torsion. Preoperative diagnosis of AH can be difficult due to its rarity and in majority diagnosis is made at operation. We hereby report a case of complicated AH in a neonate who presented atypically with a tender right inguino-scrotal swelling. Surgical exploration showed gangrenous appendix with a peri-appendicular abscess in the inguinal hernia sac. 

## CASE REPORT

A 5-day-old, term neonate was referred with a right inguino-scrotal swelling noted for one day. The baby had been irritable for last 12 hours, but still was able to tolerate feeds and have bowel movements. There was no history of fever or vomiting and birth history was unremarkable. On physical examination, the baby had dysmorphic features along with bilateral central cleft lip and palate, bilateral postaxial polydactyly and clenched fists. His abdominal examination was normal and local examination showed a firm right irreducible inguino-scrotal swelling with mild redness of the overlying skin and the right testis was not palpable separately (Fig. 1). The left inguino-scrotal examination was normal with left testis in the scrotum. His WBC (16.1X103/μl) and C-reactive protein (103 mg/L) were high. Ultrasound findings included increased vascularity of both testis and scrotal wall edema. A diagnosis of right epididymo-orchitis was considered and patient was started on IV antibiotics (Ampicillin 100mg/kg and Gentamycin 4mg/kg). In next 36-48 hours, he remained clinically stable and also his laboratory results improved, but right inguinoscrotal swelling aggravated, overlying skin showed marked redness. A repeat ultrasound revealed an elongated tubular structure within the hernia sac along with turbid fluid and debris, enlarged right epididymis and both testes were in the scrotum. Emergency surgical exploration through right inguinal incision showed a firm swelling extending from the internal ring to the right scrotum, which on opening contained dark-brownish fluid and a blackish tubular structure. Further exploration revealed mildly congested caecum and terminal ileum and black tubular structure was identified as appendix, which was twisted and gangrenous (Fig. 2). The right testicle showed enlarged epididymis and evidence of significant inflammation. Appendectomy and right inguinal herniotomy was performed. Histopathology of the resected appendix showed intense acute inflammation with focal suppuration suggestive of infarcted appendix. Genetic work up for dysmorphic features revealed mosaic Trisomy 13. Patient has been reviewed in outpatient clinic 3-months after surgery and has remained well with no complications. 

**Figure:**
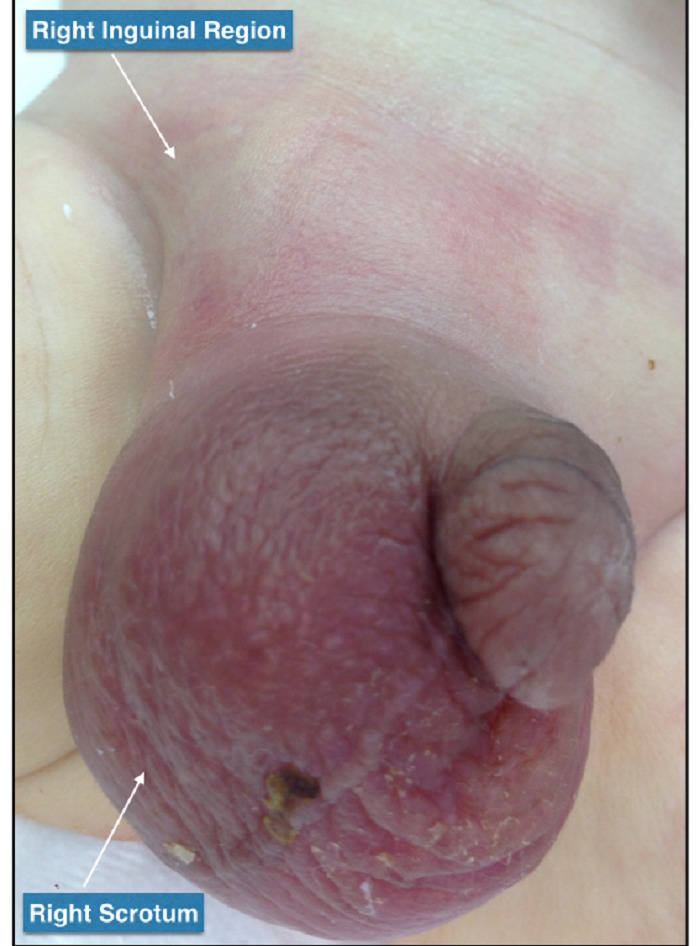
Figure 1: Preoperative images showing swollen and red right inguinoscrotal region on 3rd day of presentation. Right testis cannot be palpated separately.

**Figure:**
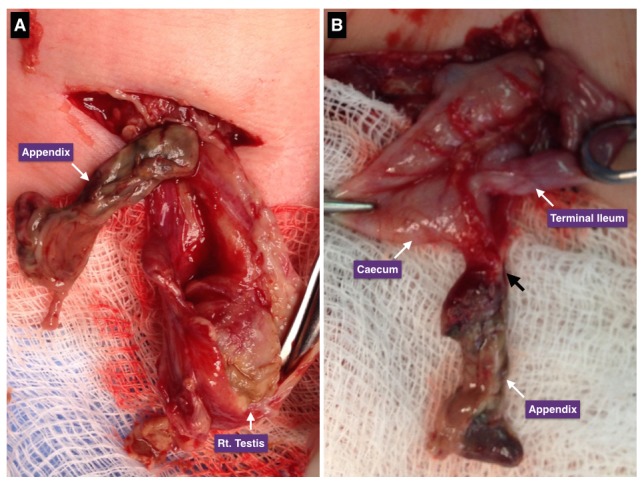
Figure 2: Surgical exploration through right inguinal incision. A). Gangrenous appendix in the right patent processus vaginalis (opened), right spermatic cord and testis are significantly inflamed B). Further exploration revealed mildly congested caecum and terminal ileum and most of the appendix was gangrenous, black arrow indicates the point where the appendix was twisted.

## DISCUSSION

When appendix is present in the hernia sac, it is termed “Amyand’s hernia” (AH); named after Claudius Amyand, surgeon of King George II, who described this first in 1735 while operating on an 11-year-old boy for inguinal hernia [2]. AH is very rare and only few cases have been reported in neonates [3, 4]. Finding an appendix complicated by acute appendicitis and peri appendicular abscess in AH is extremely rare and only 0.08% incidence has been reported [1]. Preoperative diagnosis of AH is difficult due to its rarity and the clinical findings are similar to that of any inguinal hernia. When AH is complicated by inflamed appendix and/or peri appendicular collection, the associated erythema of overlying and surrounding skin, local tenderness, and difficulty/inability to reduce the hernia contents resemble clinical picture of incarcerated inguinal hernia, epididymo-orchitis or testicular torsion. Therefore, AH is usually diagnosed during surgery. Ultrasonography may be helpful to differentiate inguinal hernias from other acute inguinoscrotal conditions [5] like in our case. In this case, the appendix showed torsion close to the caecum, which could be due to repeated attempts of manipulation to examine and/or to reduce the contents of hernia. The associated significant inflammation of right testis may be secondary to inflammation of appendix, which was sitting close to the right testis. Absence of signs of peritonitis indicates a localized inflammatory process and not extending to the bowel in peritoneal cavity. 

We propose to consider an alternative diagnosis in acute scrotum in neonates when findings are equivocal and also reinforce the practice of early surgical exploration.

The pathophysiology of acute appendicitis in AH is not clear. The proposed theories include the presence of congenital band extending from the appendix into the scrotum up to the right testis [6], incidental or due to decrease in the vascularity of appendix secondary to incarceration [7]. In our case, the appendix showed torsion close to the caecum, which could be due to repeated attempts of manipulation to examine and/or to reduce the contents of hernia. The associated significant inflammation of right testis may be secondary to inflammation of appendix, which was sitting close to the right testis. Absence of signs of peritonitis indicates a localized inflammatory process and not extending to the bowel in peritoneal cavity.

In conclusion, we emphasize the need to consider alternative diagnosis in acute scrotum in neonates when findings are equivocal and reinforce the practice of early surgical exploration.

## Footnotes

**Source of Support:** Nil

**Conflict of Interest:** None

